# Urogenital schistosomiasis is associated with signatures of microbiome dysbiosis in Nigerian adolescents

**DOI:** 10.1038/s41598-018-36709-1

**Published:** 2019-01-29

**Authors:** Olumide Ajibola, Aislinn D. Rowan, Clement O. Ogedengbe, Mari B. Mshelia, Damien J. Cabral, Anthonius A. Eze, Stephen Obaro, Peter Belenky

**Affiliations:** 1 Department of Microbiology, Faculty of Science, Federal University Birnin Kebbi, Birnin Kebbi, Kebbi State Nigeria; 2 Department of Molecular Microbiology and Immunology, Division of Biology and Medicine, Brown University, Providence, RI USA; 3 Department of Medical Biochemistry, College of Medicine, University of Nigeria - Enugu Campus, Enugu, Nigeria; 4 Division of Pediatric Infectious Diseases, University of Nebraska Medical Center, Omaha, NE USA; 5 International Foundation Against Infectious Diseases in Nigeria, Department of Pediatrics, Bayero University Kano, Kano, Nigeria; 6 Medical Research Council Unit The Gambia at London School of Hygiene and Tropical Medicine, Banjul, The Gambia

## Abstract

Urogenital schistosomiasis is a neglected tropical disease caused by the parasite *Schistosoma haematobium*, which resides in the vasculature surrounding the urogenital system. Previous work has suggested that helminthic infections can affect the intestinal microbiome, and we hypothesized that *S*. *haematobium* infection could result in an alteration of immune system-microbiota homeostasis and impact the composition of the gut microbiota. To address this question, we compared the fecal microbiomes of infected and uninfected schoolchildren from the Argungu Local Government Area of Kebbi State, Nigeria, detecting significant differences in community composition between the two groups. Most remarkably, we observed a decreased abundance of Firmicutes and increased abundance of Proteobacteria – a shift in community structure which has been previously associated with dysbiosis. More specifically, we detected a number of changes in lower taxa reminiscent of inflammation-associated dysbiosis, including decreases in Clostridiales and increases in Moraxellaceae, Veillonellaceae, Pasteurellaceae, and Desulfovibrionaceae. Functional potential analysis also revealed an enrichment in orthologs of urease, which has been linked to dysbiosis and inflammation. Overall, our analysis indicates that *S*. *haematobium* infection is associated with perturbations in the gut microbiota and may point to microbiome disruption as an additional consequence of schistosome infection.

## Introduction

Schistosomiasis, or bilharzia, is a parasitic disease that infects hundreds of millions of people each year and is endemic to various tropical regions, notably in Africa^1^. The disease is caused by infection with trematode helminths of the genus *Schistosoma*, which live and sexually reproduce in the circulatory system of human hosts. Specifically, the species *S*. *mansoni* and *S*. *japonicum* live in venules surrounding the gut, while *S*. *haematobium* lives in the vessels around the urogenital system. There, adult worm pairs produce eggs that migrate through the surrounding tissue to be excreted primarily in the feces or urine, depending on the species, with the ultimate goal of reaching freshwater sources. They then reproduce asexually in their intermediate host – freshwater snails – before infecting humans present in contaminated water, entering through the skin before migrating to the vasculature^2–7^. The disease is typically diagnosed by microscopic examination of feces or urine for the presence of schistosome eggs^3^, although some more sensitive techniques have been developed^8^. Treatment of schistosomiasis by administration of the anti-helminth drug praziquantel is the main control strategy employed in endemic areas^9^.

The pathology of the disease generally arises from immunological reactions to eggs that become lodged in the tissue surrounding the gastrointestinal or urogenital system while attempting to migrate to the gut or bladder lumen. The eggs generally provoke a T_H_2 immune response, which is characteristic of extracellular insults including helminths and their eggs, leading to granuloma formation and fibrotic lesions that can have severe long-term consequences^4,10–16^. Eventually, the immune response is down-regulated, helping to preserve host health and integrity but allowing the parasite to persist for years^4,17,18^. This altered immune state may interplay with other immune insults, reducing the effectiveness of certain vaccines and altering the course of viral, bacterial, and parasitic co-infections^4,7,19–31^. On the other hand, it may also help to reduce the prevalence or severity of autoimmune disorders, and there is research interest in the therapeutic potential of helminths or their antigens to treat inflammatory conditions^32–38^.

There is evidence that both systemic immunological changes and helminth infection specifically are associated with changes in the gut microbiota. A number of previous studies have indicated that infection with a range of helminths – including gastrointestinal nematodes, tapeworms, tissue flukes, and schistosomes – can have impacts on the composition and function of the gut microbiome, suggesting that alterations to the gut microflora may be an under-recognized side effect of helminth infection^39–50^. However, most of this work has been done in animal models or humans infected with intestinal parasites, making it difficult to separate systemic immunological changes from effects local to the intestinal niche. In contrast, while it can occasionally localize to the enteric system (particularly during heavy infection or co-infection with *S*. *mansoni*)^51,52^, *S*. *haematobium* primarily lives within the vasculature surrounding the bladder and thus provides an opportunity to study whether helminth infection can impact the microbiome indirectly via systemic immunological or other changes that may disrupt gut homeostasis. Such a link between systemic immunity and the microbiota has been recently proposed in a Ugandan cohort, in which low CD4+ T-cell counts in HIV patients were associated with significant changes in the gut microbiome^53^; additionally, several studies suggest that immunosuppression can alter the composition and function of the gut microbiota^54–57^. Therefore, we hypothesized that urogenital schistosomiasis may disturb immune-microbial homeostasis and allow for changes in the resident taxa.

In this study, we investigated the impact of *S*. *haematobium* infection on the intestinal microbiome of adolescents aged 11–15 years in the Argungu Local Government Area of Kebbi State, Nigeria. As assessed by the Nigerian Federal Ministry of Health, Kebbi State has the highest prevalence of *S*. *haematobium* infection in the country but a very low prevalence of *S*. *mansoni*, making it an ideal location to study impacts of urogenital schistosomiasis specifically^58^. Kebbi State also has a low prevalence of soil-transmitted helminths, decreasing the likelihood of coinfections^58^. We chose to focus on adolescent schoolchildren, as children and adolescents are most likely to be infected with *S*. *haematobium* due to exposure and immunological factors^38,59–65^. Additionally, detection of differences in the human microbiome can be difficult given significant variation between individuals, which can be influenced by age, sex, diet, disease states, and other conditions^66^; to help minimize some such confounding factors, we selected subjects living in the same region, attending the same school, and falling into a relatively narrow age range.

## Results

### Study Overview and Participants

In order to examine the differences in the gut microbiomes of young adolescents infected with *S*. *haematobium*, we sequenced the fecal microbiomes of 49 adolescent students: 24 individuals infected with *S*. *haematobium* and 25 controls (Table S1). A t-test indicated that the ages of the subjects do not significantly differ between the two groups (p = 0.3228), and survey data indicates that important exposure and lifestyle factors are not systematically different (Table S2). In both groups, most samples were from male students, as fewer girls attend school in the area and females are less likely to have schistosomiasis both in Kebbi State and elsewhere in Nigeria^58–61,63,67–70^. We performed analyses of community composition between male and female subjects and found no significant differences or distinct PCoA clustering (Fig. S1); therefore, males and females were grouped together for overall analyses.

In our analysis, we sequenced the V4 region of the 16S rRNA gene and were able to identify most OTUs down to the genus level using the SILVA 16S database^71^. We analyzed alpha and beta diversity in the infected and uninfected subjects, in addition to examining differences in specific taxa through computational analysis and qPCR. Finally, we used the 16S sequencing results to predict the functional potential of the infected and control gut communities.

### Metrics of Diversity Between Infection Groups

**Figure 1:**
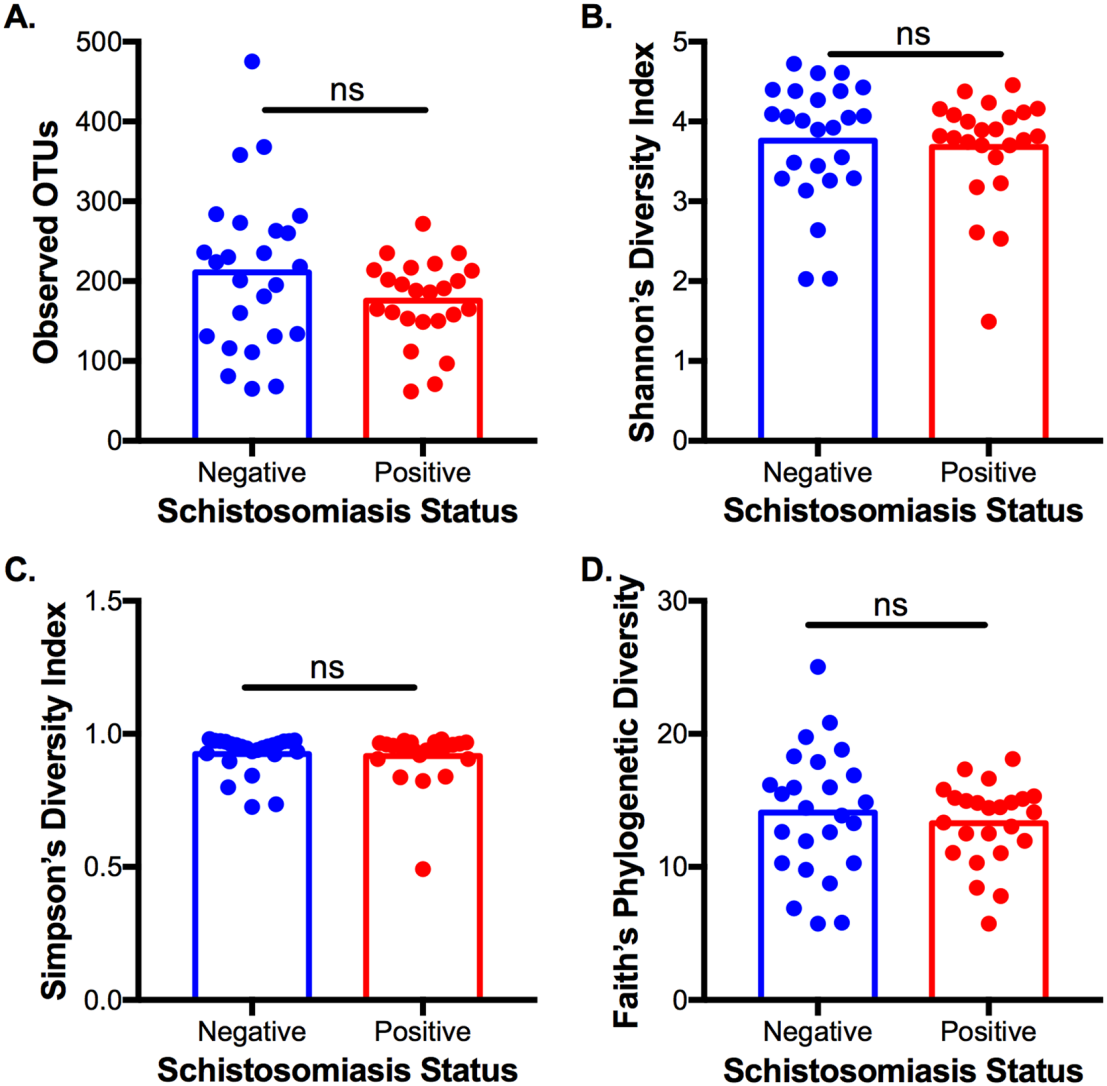
Measures of Alpha Diversity in Schistosomiasis-positive and -negative Individuals. (**A**) Observed OTUs: p = 0.12. (**B**) Shannon’s Index of diversity: p = 0.77. (**C**) Simpson’s Index of diversity: p = 0.69. (**D**) Faith’s Phylogenetic Diversity: p = 0.49. Statistics: two-tailed t-test with Welch’s correction, error bars indicate SEM.

We first examined several metrics of alpha diversity, which measures the diversity of taxa within each individual microbial community, of infected and control adolescents (Fig. 1). Observed OTUs reflects the taxonomic richness of the community (Fig. 1A), the Shannon and Simpson Diversity Indices account for both richness and abundance of taxa (Fig. 1B,C), and Faith’s Phylogenetic Diversity also considers the phylogenetic relatedness of the taxa (Fig. 1D). Using all four metrics, there was no significant difference in alpha diversity between the schistosomiasis-infected and -uninfected subjects, indicating that infection does not systematically impact the diversity of an individual’s gut microbiota.

**Figure 2:**
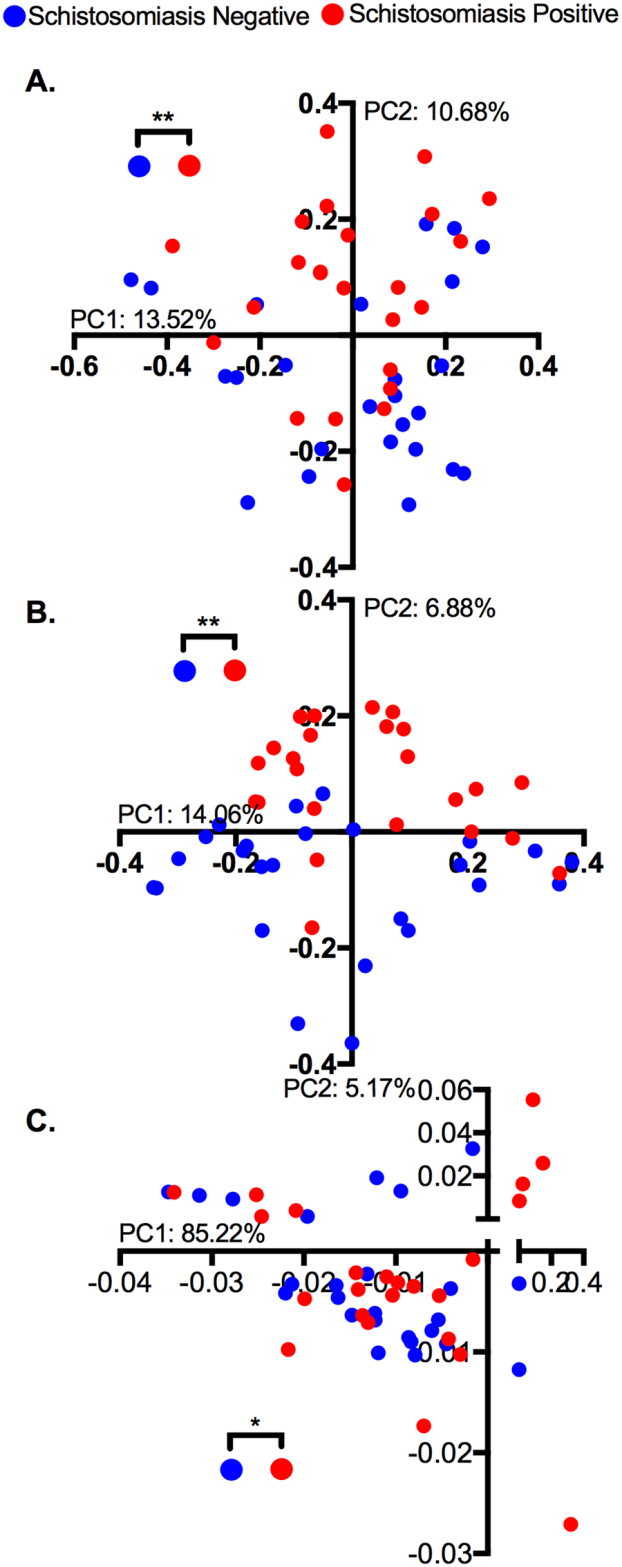
Principal Coordinate Analysis of community similarity by schistosomiasis infection status. Distance matrices were calculated using (**A**) Bray-Curtis Dissimilarity: p = 0.005. (**B**) Unweighted UniFrac: p = 0.003. (**C**) Weighted UniFrac: p = 0.012. Statistics: PERMANOVA through vegan package in R, *p < 0.05, **p < 0.01.

In contrast, we found significant differences between the microbial communities of infected and uninfected subjects when examining beta diversity, which measures the divergence in community composition between different samples. Again, we tested this using multiple metrics: Bray-Curtis dissimilarity reflects differences in the taxa present independent of their relatedness, unweighted UniFrac distance indicates differences in taxa while considering their phylogenetic relatedness, and weighted UniFrac also accounts for the abundances of the differential taxa. Using principal coordinate analyses (PCoA), we noted clustering of infected and control samples (Fig. 2) and a permutational MANOVA indicated that this difference is statistically significant in all cases. We found the greatest difference between the groups using unweighted UniFrac, suggesting that differences in community composition could be driven by changes in low-abundance taxa.

### Significantly Different Genera by Infection Status

**Figure 3:**
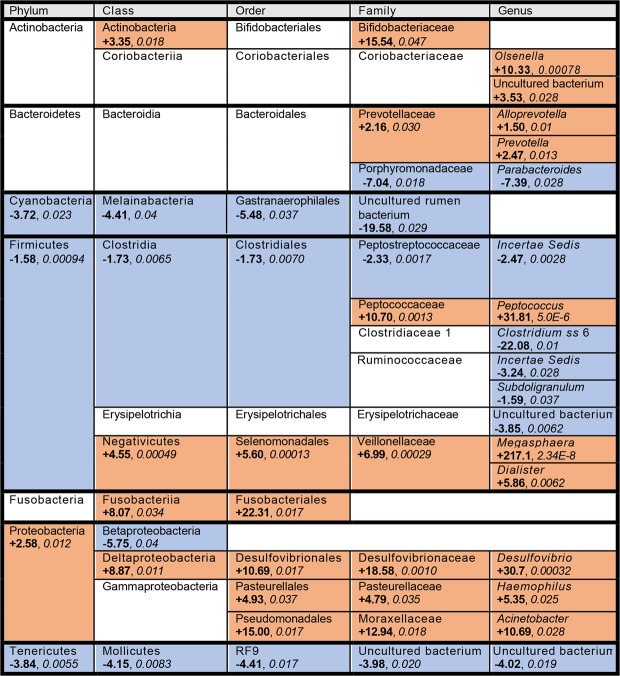
Taxa and associated lineages that significantly changed in schistosomiasis-positive subjects. Taxa that increased significantly in infected subjects are shown in orange cells, while taxa that decreased significantly are shown in blue cells. Taxa that did not change or with changes that did not reach significance are shown in white cells. Fold change values (shown in bold) were calculated from the log_2_(fold change) value output from DESeq2, and FDR values (shown in *italics*) were obtained from the same DESeq2 output.

**Figure 4:**
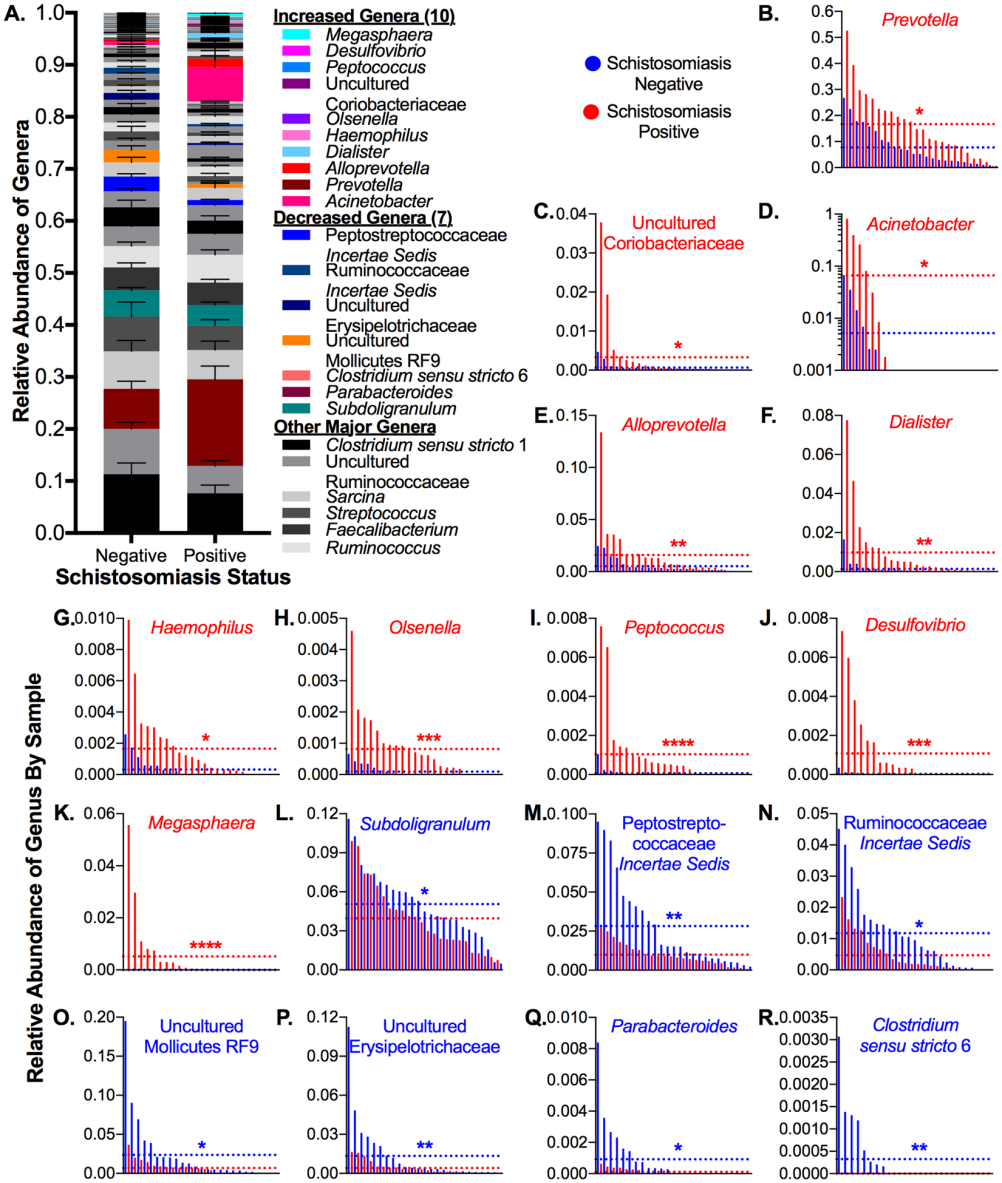
Differences in Relative Abundances of Genera Between Schistosomiasis-positive and -negative Subjects. (**A**) Average relative abundances of all genera, with genera showing significant differences between positive and negative samples highlighted in color. (**B**–**R**) Genera that changed in infected adolescents, with negative and positive samples interleaved by ranked abundance of each taxon and dotted lines representing the average relative abundance by group. Statistics: Wald test of differential abundance through DESeq2 package in R, *p < 0.05, **p < 0.01, ***p < 0.001, ****p < 0.0001, error bars indicate SEM. Exact corrected p-values (FDR) can be found in Fig. 3.

Given the significant differences in beta diversity, we examined the differential abundance of taxa between the infected and uninfected subjects. In total, 1,660 unique OTUs were identified across all samples. As most OTUs were not identified down to the species level, we agglomerated our samples at the genus level to perform differential abundance analysis. We detected significant differences in 17 genera: 10 increased (*Megasphaera*, *Dialister*, *Acinetobacter*, *Prevotella*, *Alloprevotella*, *Desulfovibrio*, *Haemophilus*, *Peptococcus*, *Olsenella*, and uncultured Coriobacteriaceae) and 7 decreased (*Subdoligranulum*, *Parabacteroides*, uncultured Erysipelotrichaceae, Ruminococcaceae *incertae sedis*, Peptostreptococcaceae *incertae sedis*, *Clostridium sensu stricto* 6, and uncultured Mollicutes RF9) in infected adolescents (Figs. 3, 4). Collectively, these 17 genera comprised an average of 23% of the relative abundance of the microbiota of uninfected subjects, and all have been previously specifically associated with or arise from lineages associated with the human gut microbiota^72–85^. Decreases were mainly found within the phylum Firmicutes, particularly in the class Clostridiales, while increases were mainly found within the phylum Proteobacteria and the family Veillonellaceae.

**Figure 5:**
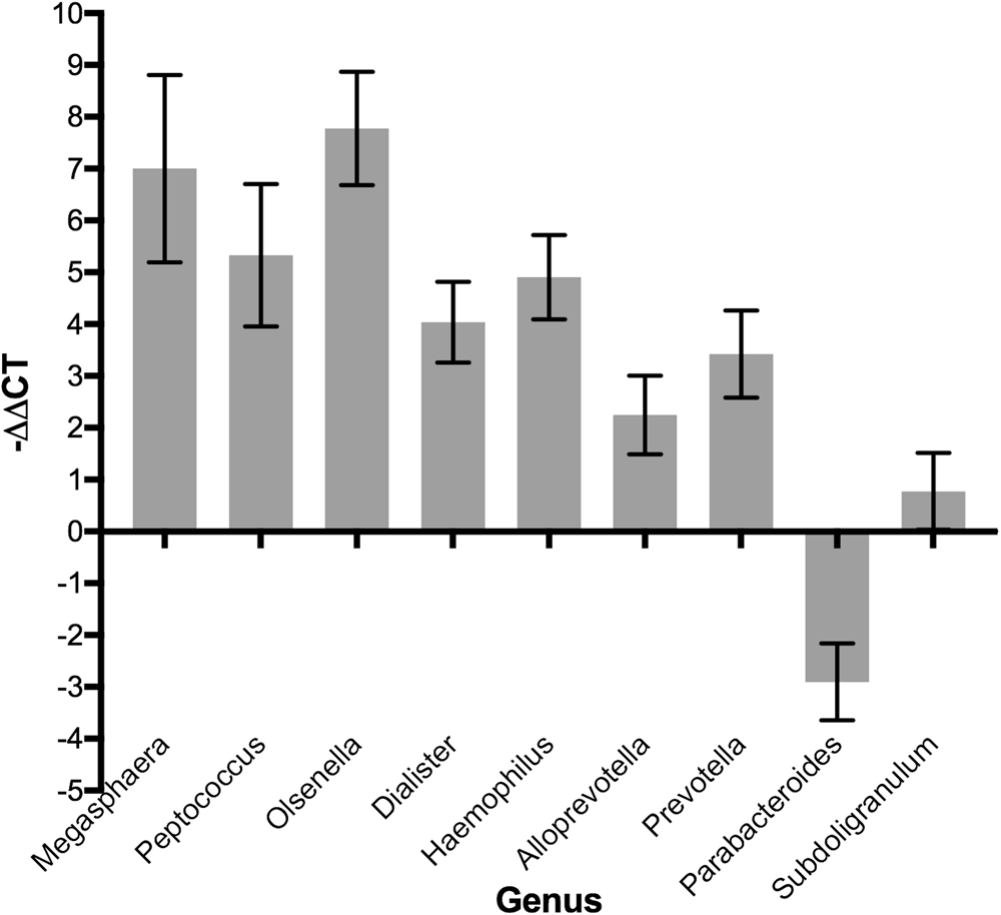
−ΔΔC_T_ Values of Significant Genera Obtained from qPCR Using Genus-specific Primers. ΔΔC_T_, used to allow all genera to be shown on the same scale, is the corrected raw difference in C_T_ values between infected and uninfected samples, and the sign change causes positive −ΔΔC_T_ values to indicate a positive fold change. Error bars indicate SEM of technical replicates.

Given that many of the genera that we found to be significant are of low abundance, we decided to use qPCR to independently verify the changes in several of these genera. We designed genus-specific 16S primers, validated their specificity against a mock community, and tested the abundances of each genus relative to total 16S rDNA present in the pooled genomic DNA of schistosomiasis-positive and -negative individuals. Despite differences in primers and methodologies between sequencing and qPCR, we were able to recapitulate differences in the abundances of *Prevotella*, *Peptococcus*, *Megasphaera*, *Olsenella*, *Dialister*, *Alloprevotella*, *Haemophilus*, and *Parabacteroides*, (Fig. 5), confirming that these genera did change in abundance in the schistosomiasis-positive individuals. For *Subdoligranulum*, which decreased very slightly in infected adolescents, qPCR did not detect a difference between the groups.

### Changes Across Taxonomic Levels

**Figure 6:**
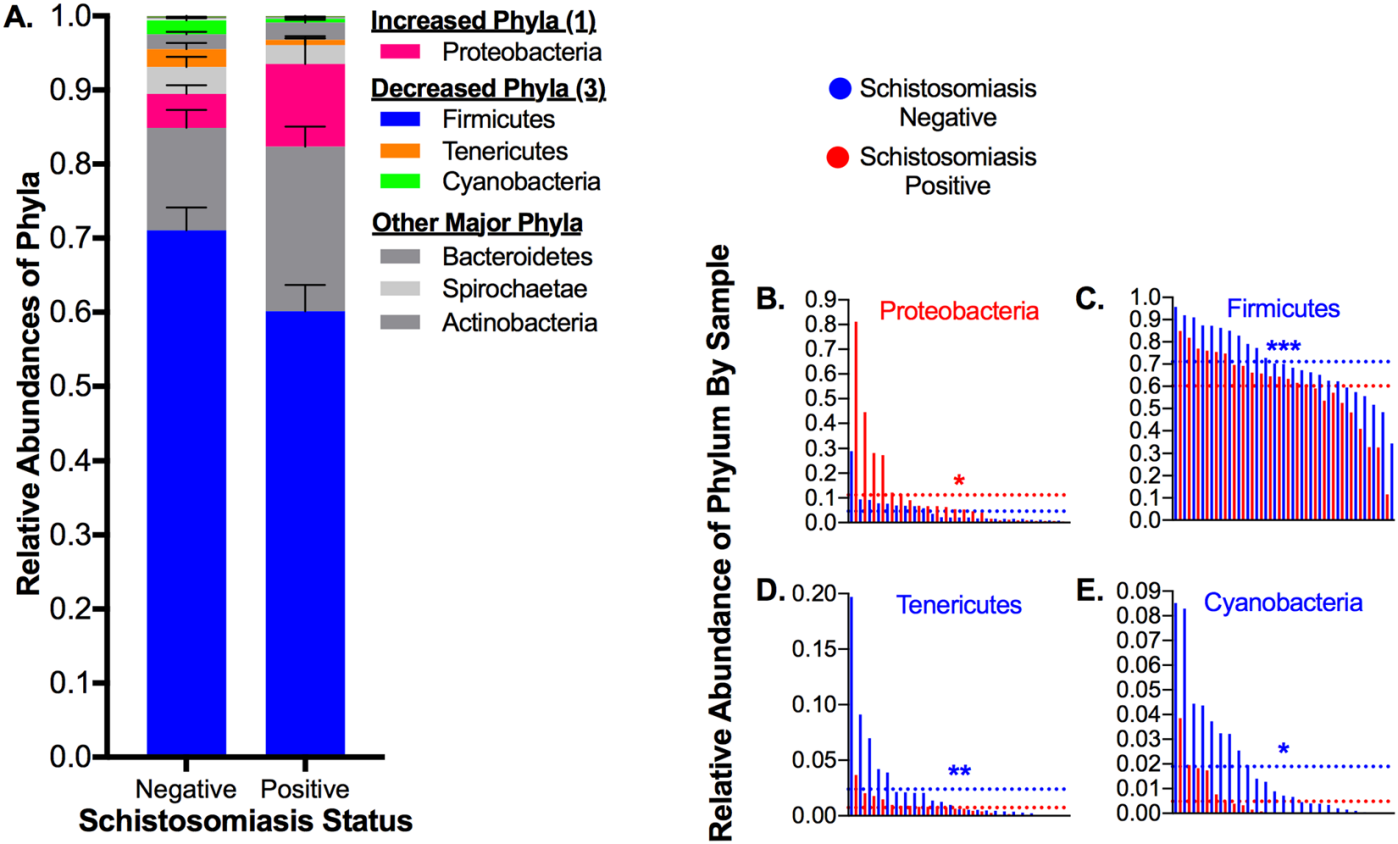
Differences in Relative Abundances of Phyla between Schistosomiasis-positive and -negative Subjects. (**A**) Average relative abundances of all phyla, with phyla showing significant differences between positive and negative samples highlighted in color. (**B**–**E**) Phyla that changed in infected adolescents, with negative and positive samples interleaved by ranked abundance of each taxon and dotted lines representing the average relative abundance by group. Statistics: Wald test of differential abundance through DESeq2 package in R, *p < 0.05, **p < 0.01, ***p < 0.001, ****p < 0.0001, error bars indicate SEM. Exact corrected p-values (FDR) can be found in Fig. 3.

We then began to look at changes in community composition at higher taxonomic levels. At the phylum level, we noted that most phyla decreased in abundance in the schistosomiasis-positive group: we observed significant decreases in Firmicutes, Tenericutes, and Cyanobacteria, and a significant increase in Proteobacteria (Figs. 3, 6). We then analyzed differential abundances at the class, order, and family levels (Figs. S2, S3, S4) and identified several lineages that show significant differences across multiple taxonomic levels (Fig. S5).

Within the phylum Proteobacteria, a number of lineages demonstrated significant increases in abundance across multiple taxonomic levels. For example, the lineage from which the genus *Desulfovibrio* arises shows significant increases across all taxonomic levels, including family (Desulfovibrionaceae), order (Desulfovibrionales), class (Deltaproteobacteria), and phylum (Proteobacteria) (Fig. S5A). However, as Deltaproteobacteria comprise a small proportion of the phylum, the increase in Proteobacteria is in fact largely driven by members of the class Gammaproteobacteria. While there was no significant difference at the class level, there were significant increases in two of its lineages: *Haemophilus*, including family Pasteurellaceae and order Pasteurellales, and *Acinetobacter*, including family Moraxellaceae and order Pseudomonadales (Fig. S5B). In the human gut, Proteobacteria are typically found at low abundances relative to the dominant phyla of Firmicutes and Bacteroidetes, but blooms in this phylum have been associated with dysbiosis^86–88^.

Similarly, there are significant increases throughout the taxonomic lineage of *Megasphaera* and *Dialister*, on the family (Veillonellaceae), order (Selenomonadales), and class (Negativicutes) levels (Fig. S5C). However, in this case, there is an overall decrease in the parent genus of Firmicutes. This may be related to the fact that Negativicutes, unlike the majority of Gram-positive Firmicutes, are diderms with distinct outer membranes containing lipopolysaccharides that cause them to stain Gram-negative^89,90^. Interestingly, it is hypothesized that these genes may have been laterally acquired from Proteobacteria^91^, which also increase in infected adolescents; it is possible that this similarity gives both groups a competitive advantage in the schistosomiasis-associated microbiota.

Additionally, while the Peptococcaceae family from which the genus *Peptococcus* stems is significantly increased, the order (Clostridiales), class (Clostridia), and phylum (Firmicutes) are significantly decreased (Fig. S5D). In fact, *Peptococcus* is the only significant genus within the Clostridiales lineage that increases in infected individuals, while the several other significant genera all decrease. For example, the related lineage of Peptostreptococcaceae *incertae sedis* shows reductions in abundance at all levels, reflecting the more typical pattern of members of Clostridiales and Firmicutes in general (Fig. S5D).

Finally, there are two lineages from less-common phyla that demonstrate reductions in abundance in schistomiasis-positive subjects: the Gastranaerophilales-Melainabacteria lineage of Cyanobacteria and the RF9-Mollicutes lineage of Tenericutes. Unlike most Cyanobacteria, the Melainabacteria are non-photosynthetic and rely on fermentation^92,93^, while Mollicutes are distinguished from most other bacteria by their lack of a cell wall^94^.

### Functional Potential of the Microbial Communities

While taxonomic classifications of the microbial communities of the two groups is useful, we were also interested in the functional potential of the gut microbiome and how it might vary between infected and uninfected individuals^95,96^. In general, while there are often significant inter-individual differences in the taxonomic composition of the gut microbiome, the functionality of the resident taxa is relatively stable^97^. Recently, methods have become available to use the 16S content of a microbial community to infer the genomes present, and therefore the potential functionality of that community, in the absence of whole-genome sequencing data^98–100^. Importantly, it should be noted that this methodology is based on inference from known genomes, and therefore may not fully recapitulate the existing metagenomic content, relevant strain differences, and the contributions of understudied microbiota members.

We used the web-based tool Piphillin to predict changes in the functional potential of the microbiome by inferring the metagenomes from 16S sequences^99^ and the tool MicrobiomeAnalyst to analyze differential abundance of both KEGG Orthologs and Pathways^101^. We identified two KEGG pathways that were enriched in schistosomiasis-positive individuals (Table S3), as well as 35 KEGG orthologs that were significantly different between the two groups (Table S4). We were particularly interested to see that the top enriched pathway, “atrazine degradation”, was populated by the three subunits of bacterial urease (ureA, ureB, and ureC). Furthermore, there were increases in the ureD, ureE, ureF, ureG, and ureH orthologs, all urease accessory proteins, although these were not categorized into any KEGG pathways. Full metagenomics, metatranscriptomics, or functional assays could determine whether the increases observed here reflect a true increase in urease production or function in these microbial communities.

## Discussion

We observed a general shift in the gut microbiome of adolescents infected with *S*. *haematobium* towards a state consistent with dysbiosis, with decreases in the dominant phylum Firmicutes and increases in the prevalence of the minor phylum Proteobacteria (Fig. 6). At the genus level, where we focused our analysis, we observed significant changes in seventeen genera collectively comprising over 20% of the gut microflora (Figs. 3, 4, Data S1). Interestingly, many of the changes we observed have been associated with gut inflammation. This was surprising, as previously helminth infection has been shown to reduce inflammation, and has even been investigated as a therapy to ameliorate symptoms of inflammatory bowel disease^34,35^. The apparent contrast could result from the distant location of *S*. *haematobium*; gut-resident helminths may exert local anti-inflammatory effects that are not observed in urogenital schistosomiasis. In general, however, these changes are consistent with our hypothesis that *S*. *haematobium* infection may impact the gut microbiota.

Several of the changes we found are similar to those observed in other studies of the microbiota of humans infected with helminths. Most directly, a study of Zimbabwean children found significant increases in several OTUs belonging to the genus *Prevotella* in *S*. *haematobium*-infected subjects^50^, a change we also observed on the genus level. Additionally, several studies investigating the impacts of gut-resident soil-transmitted helminths (STH) demonstrated some of the same taxonomic changes we observed. For example, a study in a Malaysian population infected with multiple STH found increases in the order Bacteroidales^40^. Similarly, we observed increases in the *Prevotella* and *Alloprevotella* genera within Bacteroidales, although we also observed a decrease in the *Parabacteroides* genus in this order. Another study found increases in *Olsenella* and the *Desulfovibrio* lineage in individuals infected with STH in both Indonesian and Liberian populations, as well as associations between STH infection and the *Dialister* lineage in the Indonesian group and *Megasphaera* and *Peptococcus* in the Liberian group^39^. However, this study also generally found increased abundances of Clostridiales members associated with helminths, which is largely contrary to what we observed^39^. Finally, researchers studying an Ecuadorean population generally found minimal differences in the microbiota of children with STH infections, but did find significant reductions in members of Clostridiales in children with mixed *Trichuris trichiura* and *Ascaris lumbricoides* infections^41^.

In addition to similarities with other studies of helminths and the human microbiome, we noted that some of the changes we observed were reminiscent of those seen in dysbiosis and inflammation. On the phylum level, decreases in the prevalence of Firmicutes have previously been associated with gut inflammation^86,102,103^. Firmicutes typically make up a significant proportion of the human gut microbiota, and some members are associated with immunoregulatory impacts. Firmicutes – particularly Clostridia – are associated with regulatory T-cell activation^104–106^, which is important for the prevention of intestinal inflammation. Additionally, some members of the phylum – such as *Faecalibacterium prausnitzii*, a member of the Clostridiales-Ruminococcaceae lineage – have been shown to have anti-inflammatory effects due to production of the short-chain fatty acid butyrate and have been negatively correlated with inflammatory bowel disease^107^.

Furthermore, increased levels of Proteobacteria have been associated with gut inflammation in a number of studies^86–88^, although whether they are causative or symptomatic remains unclear. Inflammation is associated with increased levels of oxygen and production of nitrate by the gut epithelium; Proteobacteria are generally aerotolerant and some have the capacity to utilize nitrate, potentially allowing them to outcompete other members of the microbiota – such as Clostridia – and bloom during inflammatory conditions^87,88,108,109^. In addition to thriving in an inflammatory environment, Proteobacteria themselves may contribute to inflammation. Relevant to our study, *Desulfovibrio* and other sulfate-reducing bacteria have been associated with gut inflammation and colitis, potentially through their production of cytotoxic hydrogen sulfide^110–115^. Additionally, Gram-negative bacteria, such as Proteobacteria and Negativicutes, can exacerbate existing gut inflammation through lipopolysaccharide infiltration into circulation^116,117^. Finally, it was recently shown that urease producers, potentially enriched in schistosomiasis-infected subjects, may contribute to a dysbiotic environment, favoring Proteobacteria at the expense of Clostridia and potentially promoting inflammation through increased nitrogen flux^118^.

In addition to these phylum-level shifts, we observed some changes on lower taxonomic levels that were also associated with gut inflammation. A large study of the gut mucosal and stool microbiota in new-onset pediatric Crohn’s disease patients revealed a number of changes that were similar to what we observed, including reductions in the order Clostridiales and the family Erysipelotrichaceae and increases in the families Veillonellaceae and Pasteurellaceae in patients with disease^119^. Similarly, we saw reductions in Clostridiales and a genus within Erysipelotrichaceae and increases in both Veillonellaceae and Pasteurellaceae*;* the exception is *Peptococcus*, which we saw increased within the Clostridiales family (Fig. 3). Additionally, researchers observed reductions in the order Bacteroidales and increases in the family Fusobacteriaceae in the Crohn’s disease patients^119^; we noted both increases (*Prevotella* and *Alloprevotella* within Prevotellaceae) and decreases (*Parabacteroides* within Porphyromonadaceae) within Bacteroidales in infected subjects, as well as increases in higher taxonomic levels (Fusobacteriia, Fusobacteriales) of the Fusobacteriaceae lineage (Fig. 3).

In another study, researchers compared the microbiota associated with inflamed mucosa with normal tissue in ulcerative colitis patients, finding that inflamed mucosa was enriched in Proteobacteria and reduced in Firmicutes. Furthermore, these changes were driven largely by increases in the abundance of the Pseudomonadales-Moraxellaceae-*Acinetobacter* lineage of Proteobacteria and decreases in the Clostridia-Clostridiales lineage, particularly Ruminococcaceae^120^. Reductions in Ruminococcaceae have also been observed in other studies of inflammatory bowel disease^121,122^. This is quite similar to our observations in the microbiota of schistosomiasis-infected adolescents, where we saw an enrichment in the *Acinetobacter* lineage (Figs. 3, S5B) and reductions in many members of Clostridiales, including two Ruminococcaceae (*Subdoligranulum* and an *incertae sedis*) (Fig. 3). Taken together, these results suggest that the gut microbiota of *S*. *haematobium*-infected adolescents may reflect an inflammatory environment.

Importantly, it should be noted that while some of our observations have been seen in inflammation-related contexts in other individuals, they are not diagnostic of inflammation and it is unknown whether urogenital schistosomiasis-infected adolescents actually experience intestinal inflammation. In the future, it may be prudent to profile gut inflammation in this population in conjunction with microbiome analysis, potentially through measuring fecal biomarkers such as calprotectin^123^. If, in fact, there is intestinal inflammation associated with schistosomiasis and microbiome alterations, the directionality of this effect would remain unclear; infection-mediated immunological shifts might allow a bloom of pro-inflammatory microflora or might cause inflammation that allows dysbiotic microbes to proliferate.

Additionally, a potential confounder is the presence of co-infection with enteric helminths. While we selected our region of study due to its low rates of these infections^58^ and ruled out subjects with gastrointestinal symptoms, we also used PCR to check for the presence of these organisms in extracted fecal DNA. Using previously published species-specific primers^124,125^, we did not detect *Ascaris* spp, *Ancyclostoma* spp, *Necator americanus*, *Trichuris trichiura*, or *S*. *mansoni* in samples from either infected or uninfected subjects (Fig. S6). Additionally, while *S*. *haematobium* primarily excretes eggs through the bladder, in a small percentage of cases it can also take up residence in the enteric system and extrude eggs through the intestinal wall^51,52^. Therefore, we also used PCR to check for the presence of *S*. *haematobium* in the fecal samples, and did not detect it in samples from either group (Fig. S6). Importantly, while more sensitive than microscopic methods such as Kato-Katz^8,126^, even PCR is not perfectly sensitive on a single stool sample for detection of infection with gastrointestinal helminths or schistosomes; thus, while unlikely, it is still possible that some subjects may have those underlying infections and that a portion of our dysbiotic signal may originate from such intestinal morbidity.

Additionally, while we hypothesized that *S*. *haematobium* infection could lead to alterations in the gut microbiota due to its impacts on immune function, we cannot discount the possibility that adolescents with pre-existing dysbiosis may be more susceptible to successful schistosome infections, potentially due to immunological changes mediated by the gut microflora. For example, there is evidence that the gut microbiome influences the course of infection with *S*. *mansoni*, potentially via immunoregulatory effects; abolishing the gut microbiome of mice infected with *S*. *mansoni* reduces gut inflammation and egg excretion, although this may be due to local interactions as this parasite lives proximal to the gut itself^127^. It is even possible that the “uninfected” microbiome reflects the status of individuals who have acquired immunity to reinfection. In this observational study, it is not possible to determine whether *S*. *haematobium* infection is antecedent to changes in gut microbiota. We envision that a longitudinal study of the microbiome of children in schistosomiasis-endemic areas that profiles the same individuals before, during, and after clearance of *S*. *haematobium* infection via praziquantel treatment, could help to elucidate cause and effect in the system. In addition, our study was relatively small and subjects were recruited from a single site. It would be prudent to replicate our results in a larger, multi-site study to determine whether our findings are applicable to a wider community. Similarly, profiling the microbiota in a younger cohort may also be sensible, as the potential for gut inflammation could contribute to malnutrition and growth inhibition observed in infected children^128^.

In general, we have found that the adolescent gut microbiome may be shifted towards a dysbiotic state by infection with *S*. *haematobium*, with some similarities to prior observations of the gut microbiota in inflammatory contexts. Such a broad dysbiosis would be an interesting observation in urogenital schistosomiasis, building on the increased abundance of *Prevotella* OTUs associated with infection previously observed by Kay *et al*.^50^. Given the endemic nature of infection in tropical and subtropical regions, it is important to assess how potential dysbiosis may contribute to disease morbidity. In particular, infected adolescents should be assessed for the presence of intestinal inflammation to determine whether these observed microbiome changes truly reflect an inflammatory state or are associated with any of the known morbidities of urogenital schistosomiasis. Additionally, even the schistosomiasis-negative individuals in the current study are likely to have been infected in the past, due to the endemic nature of the infection, but their microbiomes were significantly different than those of currently-infected individuals. Therefore, it would be useful to track subjects long-term after curative praziquantel administration to see whether and how quickly their microbiota returns to an uninfected state; this would additionally help to clarify the causality of the observed changes.

## Methods

### Ethics Statement

This study was approved by the Kebbi State Ministry of Health and permission to visit the Gotomo Primary School in the Argungu Local Government Area was obtained from the local government education department. All research was undertaken in accordance with the the relevant guidelines and regulations of the Kebbi State Ministry of Health. The study and its risks were explained to the students, who then verbally assented to participation if they were interested. To reduce the risk of co-infection with gastrointestinal helminths, any potential subjects who reported recent gastrointestinal distress were excluded. Prior to sample collection, the parents/guardians of students who had assented to participate were informed of the study as well as the associated risks. Parents of assented students gave informed approval for their child’s participation by signing the study consent form; in the case of illiteracy, thumbprinting was used, as approved by the State Ministry of Health Ethics Committee. All identified cases of schistosomiasis were reported to the Department of Neglected Tropical Diseases at the Kebbi State Ministry of Health. All children at the school were treated with 40 mg/kg praziquantel by the state government as part of the routine national schistosomiasis control program within two weeks after our study.

### Subject Characteristics

All samples were collected from Gotomo Primary School, which draws students from seven local villages in rural Nigeria. Six of the villages are within 1.5 km of the school, while the seventh is within 4 km; as is common in this rural area, most students walk to the school. Compared to other Nigerian states, this area is less developed and less influenced by Western culture and diet; the communities surrounding the school is primarily small-scale farmers of low socioeconomic status. As a result, the residents of this area share similar lifestyle and dietary characteristics, which reduces potential confounders compared with more developed regions of the country.

90 subjects were screened for *S*. *haematobium*, with 40 (44.4%) identified as infected. 50 adolescents aged 11–15 years were included in the study (Table S1). All samples were collected between July and August 2017. Questionnaires were administered to all participants, covering questions on demographics including age, biological sex, maternal occupation, drinking water source, and exposure to river water (Table S2). All biological samples collected were immediately transported to the Federal University Birnin Kebbi Microbiology Laboratory for processing.

### Sample Processing

Urine samples were collected between 10 AM and 2 PM in labelled sample containers and placed in black polyethylene bags. The sedimentation technique was applied for examination of *S*. *haematobium* eggs in the urine. A minimum of 7 mL of urine was collected per subject. Urine was spun down at 1,000 × *g* for 5 minutes, the supernatant was decanted, and the sediments were examined by an experienced technician under the 40X objective of a brightfield microscope (Olympus, USA) to identify *S*. *haematobium* eggs, which are characterized by a terminal spine. The number of eggs in each sample was divided by the provided volume and multiplied by 10 to obtain normalized counts of eggs/10 mL (Table S1). The presence of eggs in the urine was used to identify cases of adolescents with urogenital schistosomiasis; for subjects with no eggs in the urine, a second sample was obtained and assessed the following day by a different technician to confirm the lack of eggs and reduce the risk of false negatives. The first 25 samples collected in each group were used in the study; cases and controls were not otherwise matched.

Stool samples were also collected from each child that provided a urine sample; at the school, stool was delivered on sterile paper, collected with sterile plastic spatulas, and stored in sterile bottles. All samples were frozen at −20 degrees Celsius within one hour of production until DNA extraction. Microbial DNA was extracted from stool samples of 25 adolescents infected with urinary schistosomiasis and 25 uninfected controls. 1 g of stool was removed from the center of defrosted fecal samples and was processed following the manufacturer’s protocol using the ZR Fungal/Bacterial DNA Kit™ ZR D6005 (Zymo Research), which utilizes robust mechanical lysis. All samples were extracted at once using the same kit. Extracted DNA was shipped frozen to Brown University for 16S sequencing.

While the prevalence of confounding co-infections in Kebbi State is low^58^, species-specific PCR was nevertheless used to help rule out the presence of gastrointestinal helminths in our subjects. We also used PCR to assess whether *S*. *haematobium* eggs were present in the stool, which can occasionally occur due to unusual placement of adult worms. Extracted fecal DNA was pooled in equimolar amounts by infection status, and PCR for *S*. *mansoni*, *S*. *haematobium*, *Ascaris* species, *Ancyclostoma* species, *T*. *trichiura*, and *N*. *americanus* was performed using previously published primers (Fig. S6)^124,125^. For positive controls, parasite genomic DNA was used; *S*. *mansoni* and *S*. *haematobium* DNA was obtained from BEI Resources, while *A*. *lumbricoides*, *A*. *duodenale*, *T*. *trichiura*, and *N*. *americanus* DNA was graciously gifted to us by the Williams Laboratory at Smith College. Primer sequences and PCR conditions are listed in Table S5.

### 16S Sequencing

Extracted genomic DNA was quantified using a Qubit2 (Invitrogen) to ensure sufficient quantity for amplification. Amplification was performed in triplicate according to the Earth Microbiome Project protocols^129^, using a library of barcoded adaptor primers (515F) and a single reverse primer (806R) to amplify the V4 region of the 16S gene (Table S6)^130^. 240 ng of each amplicon was pooled together for sequencing.

Sequencing was performed on the Illumina MiSeq platform using the paired-end 2 × 250 bp protocol. Sample 42 was of poor quality, producing only 14 reads and was therefore removed from further analysis. This left 49 samples for analysis. A total of 1,939,065 reads were obtained across all samples, with a t-test (Prism 7) indicating no significant difference in read depth between infected and uninfected samples (Table S7, p = 0.112).

### Data Processing

Reads were demultiplexed using the idemp utility, allowing for 2 barcode mismatches. Demultiplexed reads were imported into the software package Quantitative Insights Into Microbial Ecology 2 (QIIME2 version 2017-8)^131,132^. Within QIIME2, sequences were quality-filtered and denoised using the Divisive Amplicon Denoising Algorithm 2 (DADA2) pipeline^133^. A total of 1,660 ribosomal sequence variants (RSVs) were identified across all samples. Taxonomy was assigned using the 99% identity SILVA (release 119) V4 classifier^71^. RSVs are analogous to the Operational Taxonomic Units (OTUs) generated through traditional clustering methods, and we have used this more familiar terminology throughout the paper. The OTU table, rooted phylogenetic tree, representative sequences, and metadata from QIIME2 were then exported for further analysis in R (V3.3.1). Demultiplexed reads, metadata, and code are available from the Brown Digital Repository (10.7301/Z0K35RVK, https://repository.library.brown.edu/studio/item/bdr:698310/).

### Diversity Analyses

Alpha diversity metrics were calculated using the phyloseq (V1.19.1) package (Shannon and Simpson Diversity indices and Observed OTUs) and btools (V0.0.1) packages (Faith’s Phylogenetic Diversity)^134,135^. Two-tailed Welch’s t-tests (Prism 7) were used to determine the significance of differences in alpha diversity between infection groups. Rarefaction curves were generated to ensure that potential differences in OTU counts were not attributable to increased read depth (Fig. S2). Beta diversity (Bray-Curtis dissimilarity and weighted and unweighted UniFrac) was analyzed using the VEGAN (V2.4-4) package^136^, and PERMANOVA was used to analyze the significance of differences in beta diversity. Principal coordinate analysis (PCoA) was then performed on the beta diversity distance matrices to visualize any relationships between microbiome composition and infection status.

### Differential Abundance Analyses

To reduce noise, data was trimmed to include only genera that were present in at least two samples. Differential abundance analysis between infection groups was conducted using the DESeq2 (V1.14.1) package in R with agglomeration at various taxonomic levels^137^. To account for multiple hypothesis testing, a Benjamini-Hochberg correction was applied to obtain the false discovery rate (FDR), and taxa with FDR values below 0.05 were considered significant. Abundances by sample at all taxonomic levels are provided in Data S1.

### Inferred Metagenomics

To predict the functional potential of the positive and negative communities, we used the web-based tool Piphillin, which infers metagenomes from 16S content^99^ and matches them to orthologs and pathways from the Kyoto Encyclopedia of Genes and Genomes (KEGG) database. The OTU abundance table and representative sequences file were exported from QIIME2 and uploaded to Piphillin with required formatting, and the analysis was run using the following parameters: Database – KEGG; Database Version – KEGG May 2017; % Identity Cutoff – 99. The Features output from Piphillin was formatted and uploaded to the web-based tool MicrobiomeAnalyst to identify differentially abundant KEGG orthologs and pathways^101^. The following MicrobiomeAnalyst parameters were used to analyze differential abundance in orthologs: Data filtering – None; Data Normalization – Relative Log Expression; Analysis Overview – RNASeq Methods (DESeq 2). To assess pathway enrichment, the Network Mapping function on the DESeq2 output was used. In both cases, a FDR less than 0.05 was considered significant.

### Confirmatory qPCR of Specific Genus-Level Changes

Genus-specific primers were used to confirm that the changes seen in differential abundance analysis were reflected in the original templates. Many of the significant genera were taxonomically classified as “uncultured” or “*incertae sedis*” members of a higher taxonomic level, and therefore these were excluded. To design genus-specific primers, at least one 16S sequence from each of several major species in each significant genus was downloaded from the National Center for Biotechnology Information, as well as 16S sequences of representative species from other genera in the same family. When a large number of other genera were present, those also present in the samples were prioritized. For each genus, the relevant species were aligned using Muscle in UGENE (V1.28)^138^. Alignments were visually scanned for regions where the species within the relevant genus were very similar but were different from species in related genera.

After selecting a few potential regions, primer pairs were tested in NCBI’s Primer-BLAST, which combines Primer3 and BLAST^139–141^. Briefly, the 16S sequence of a representative species from the relevant genus was used as the template, and the potential primer pairs were input with the following parameters: Search Mode – Automatic; Database – Refseq Representative Genomes; Organism – Bacteria (taxid:2); Primer must have at least **3** total mismatches to unintended targets; At least **2** mismatches within the last **5** bps; Ignore targets that have **6** or more mismatches to the primer. Only primer pairs with unintended targets that matched with other species in the genus but not with species in related genera present in the sample were accepted. Primer pairs were validated by robust amplification from schistosomiasis-positive gDNA samples compared to a mock community that did not contain the genera of interest (ZymoBIOMICS Microbial Community DNA Standard). The exception is *Desulfovibrio*, which despite apparent strong specificity in PrimerBLAST did show some amplification from the mock community. Primers are listed in Table S7.

Equivalent amounts of genomic DNA from all schistosomiasis-positive and schistosomiasis-negative samples were pooled into two samples for analysis. All qPCR was run on a Roche Lightcycler 480, using the SYBRGreen-based 2X Fast Start Essential DNA Green Master Mix in the following preparation: 7.5 µL Master Mix, 6.35 µL H2O, 0.075 µL each primer, and 1 ng of template gDNA. Each qPCR run was performed in triplicate technical replicates on pooled positive gDNA, pooled negative gDNA, a negative control mock community, and a no template control. Reactions were performed in parallel. Cycling conditions are listed in Table S8. Changes were calculated using the ΔΔCT method, using total 16S DNA amplified from the pooled samples with universal primers to normalize data.

## Electronic supplementary material

Supplementary Information

Dataset 1
