# Supplementary material for: Urogenital schistosomiasis is associated with signatures of microbiome dysbiosis in Nigerian adolescents
Source: Sci Rep. 2019 Jan 29;9:829. doi: 10.1038/s41598-018-36709-1 (PMC6351658; doi:10.1038/s41598-018-36709-1)
Supplement: Supplementary file 1 — Supplementary Information [file 41598_2018_36709_MOESM1_ESM.pdf]

Supplementary Information for:

Urogenital schistosomiasis is associated with signatures of microbiome dysbiosis in Nigerian adolescents

Olumide Ajibola[1¶*], Aislinn D. Rowan[2¶], Clement O. Ogedengbe[3], Mari B. Mshelia[1], Damien J. Cabral[2], Anthonius A. Eze[3], Stephen Obaro[4,5], & Peter Belenky[2*]

[1] Department of Microbiology, Faculty of Science, Federal University Birnin Kebbi, Birnin Kebbi, Kebbi State, Nigeria
[2] Department of Molecular Microbiology and Immunology, Division of Biology and Medicine, Brown University, Providence, RI, USA
[3] Department of Medical Biochemistry, College of Medicine, University of Nigeria - Enugu Campus, Enugu, Nigeria
[4] Division of Pediatric Infectious Diseases, University of Nebraska Medical Center, Omaha, NE, USA
[5] International Foundation Against Infectious Diseases in Nigeria, Department of Pediatrics, Bayero University Kano, Kano, Nigeria

*Corresponding author
peter_belenky@brown.edu (PB), olumide.ajibola@fubk.edu.ng (OA)

¶ These authors contributed equally to this work.

Figure S1: Principal Coordinate Analysis of Community Similarity by Gender.
Distance matrices were calculated using (A) Bray-Curtis (p=0.853), (B) unweighted UniFrac (p=0.589), and (C) weighted UniFrac (p=0.53). Statistics: PERMANOVA through vegan package in R.

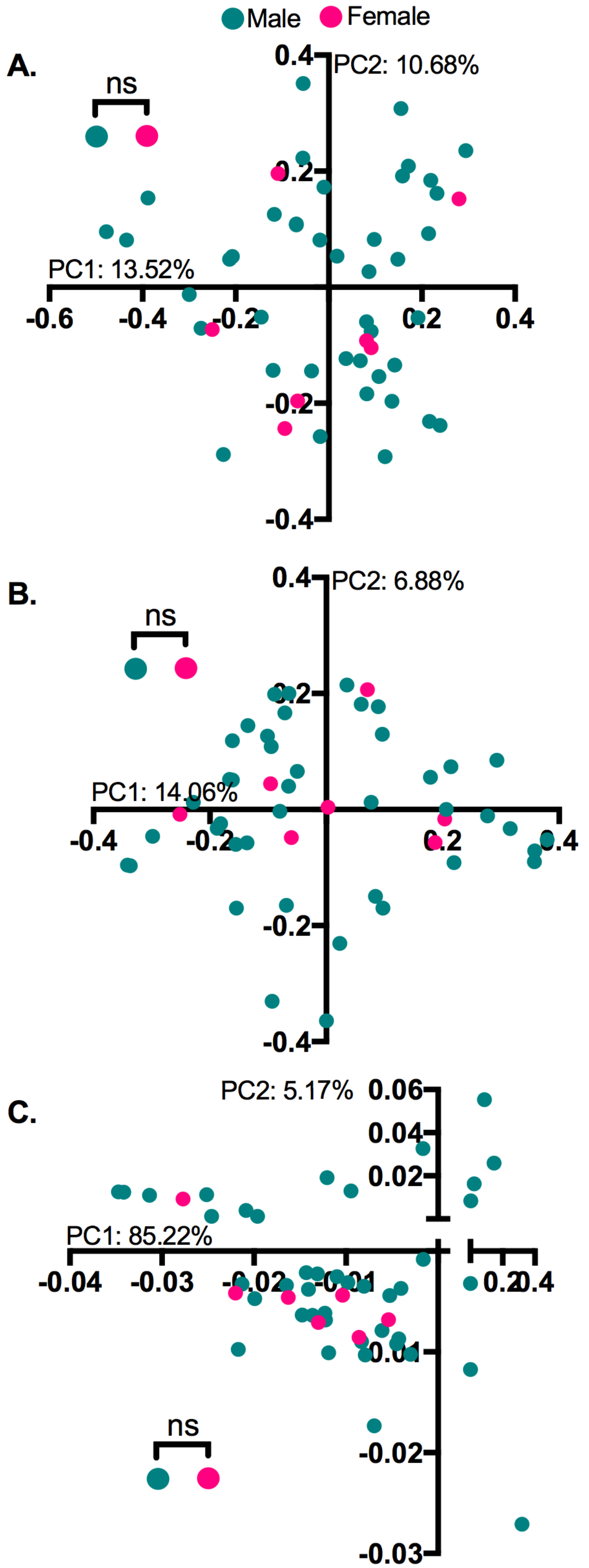

Figure S2: Differences in Relative Abundance of Classes between Schistosomiasis-positive and -negative Subjects.

(A) Average relative abundances of all classes, with classes showing significant differences between positive and negative samples highlighted in color. (B-I) Classes that changed in infected adolescents, with negative and positive samples interleaved by ranked abundance of each taxon and dotted lines representing the average relative abundance by group. Statistics: Wald test of differential abundance through DESeq2 package in R, * p<0.05, ** p<0.01, *** p<0.001, **** p<0.0001, error bars indicate SEM. Exact corrected p-values (FDR) can be found in Figure 3.

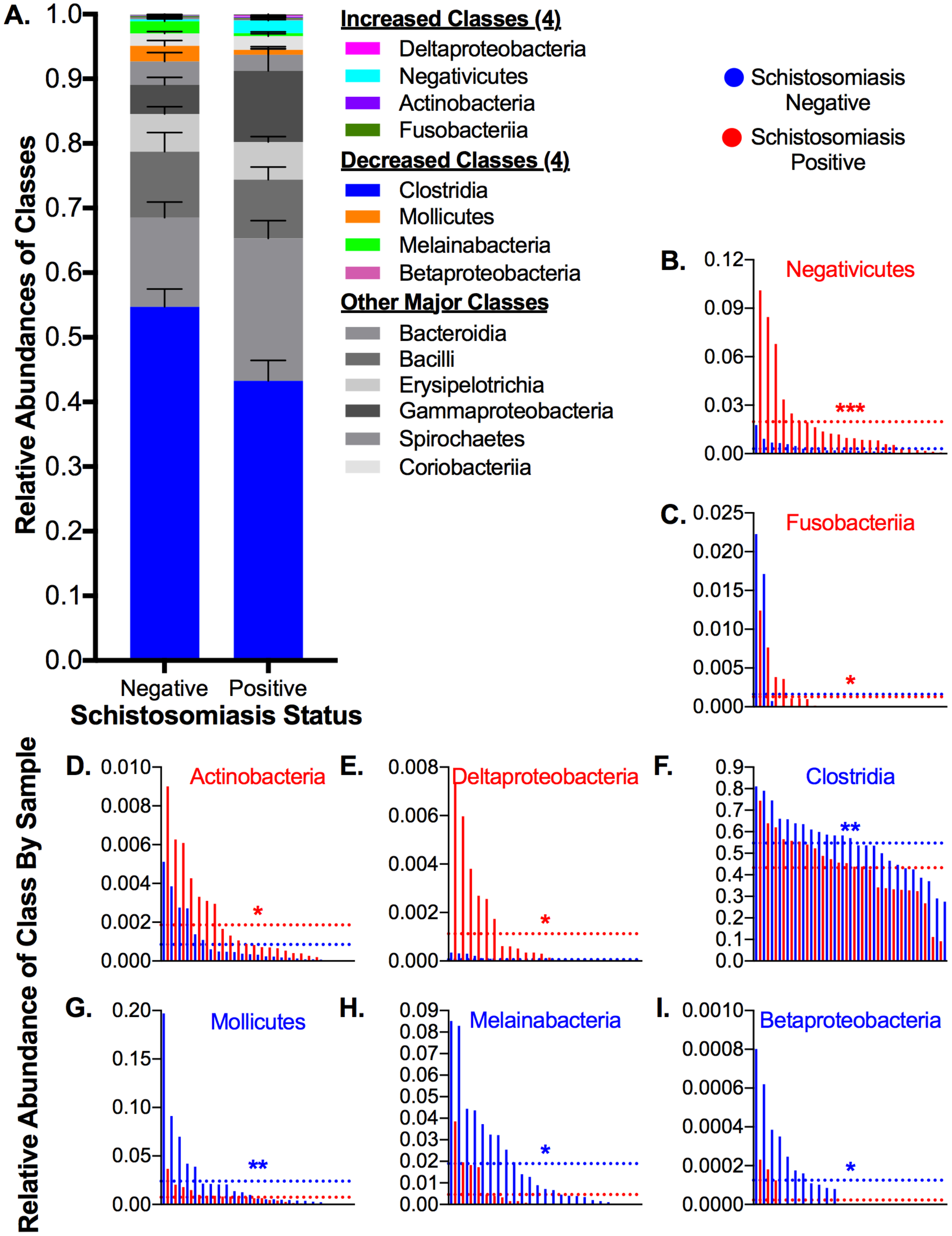

Figure S3: Differences in Relative Abundance of Orders between Schistosomiasis-positive and -negative Subjects.

(A) Average relative abundances of all orders, with orders showing significant differences between positive and negative samples highlighted in color. (B-I) Orders that changed in infected adolescents, with negative and positive samples interleaved by ranked abundance of each taxon and dotted lines representing the average relative abundance by group. Statistics: Wald test of differential abundance through DESeq2 package in R, * p<0.05, ** p<0.01, *** p<0.001, **** p<0.0001, error bars indicate SEM. Exact corrected p-values (FDR) can be found in Figure 3.

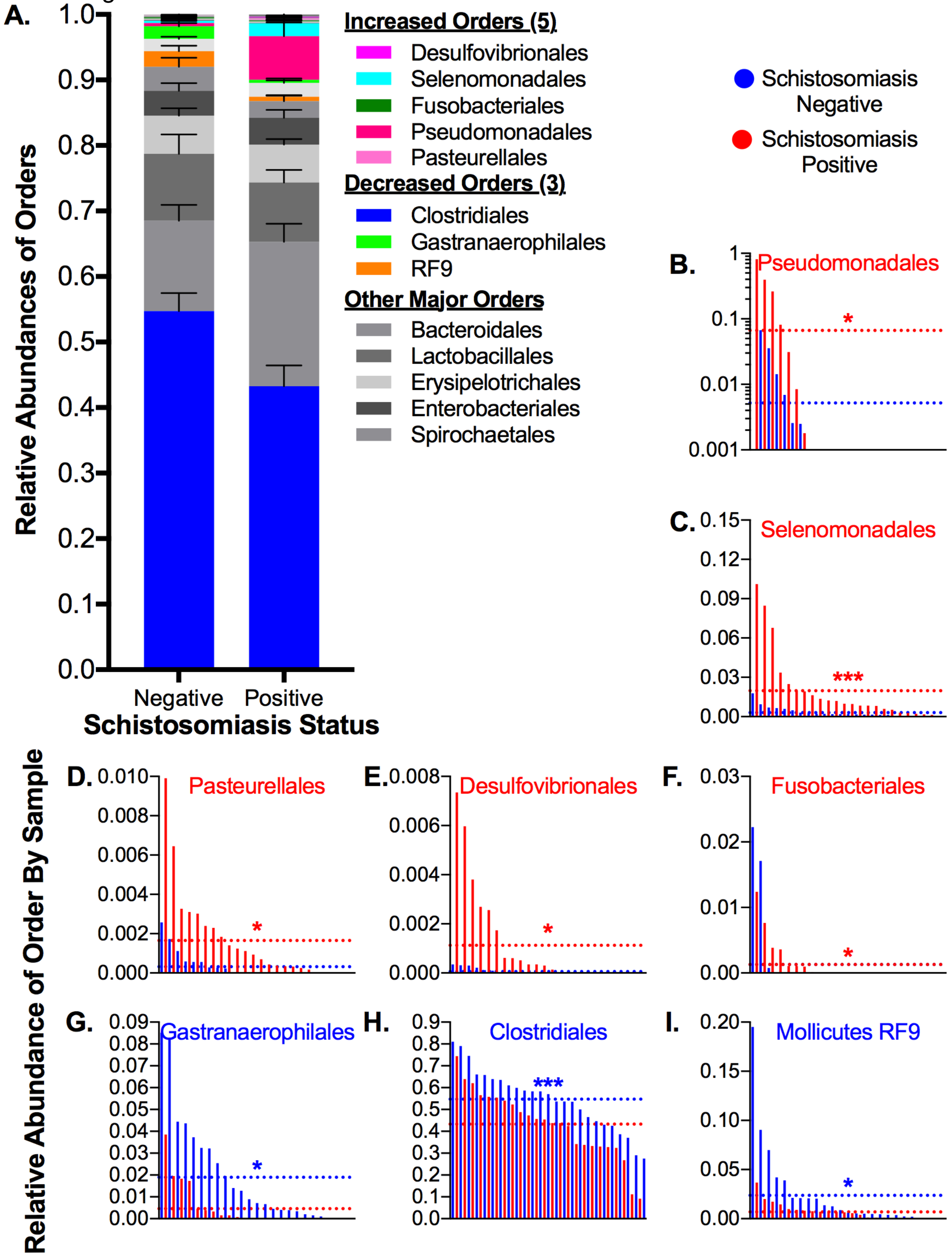

Figure S4: Differences in Relative Abundance of Families between Schistosomiasis-positive and -negative Subjects.
(A) Average relative abundances of all families, with families showing significant differences between positive and negative samples highlighted in color. (B-L) Families that changed in infected adolescents, with negative and positive samples interleaved by ranked abundance of each taxon and dotted lines representing the average by group. Statistics: Wald test of differential abundance through DESeq2 package in R, * $p<0.05$, ** $p<0.01$ error bars indicate SEM. Exact corrected p-values (FDR) can be found in Figure 3.

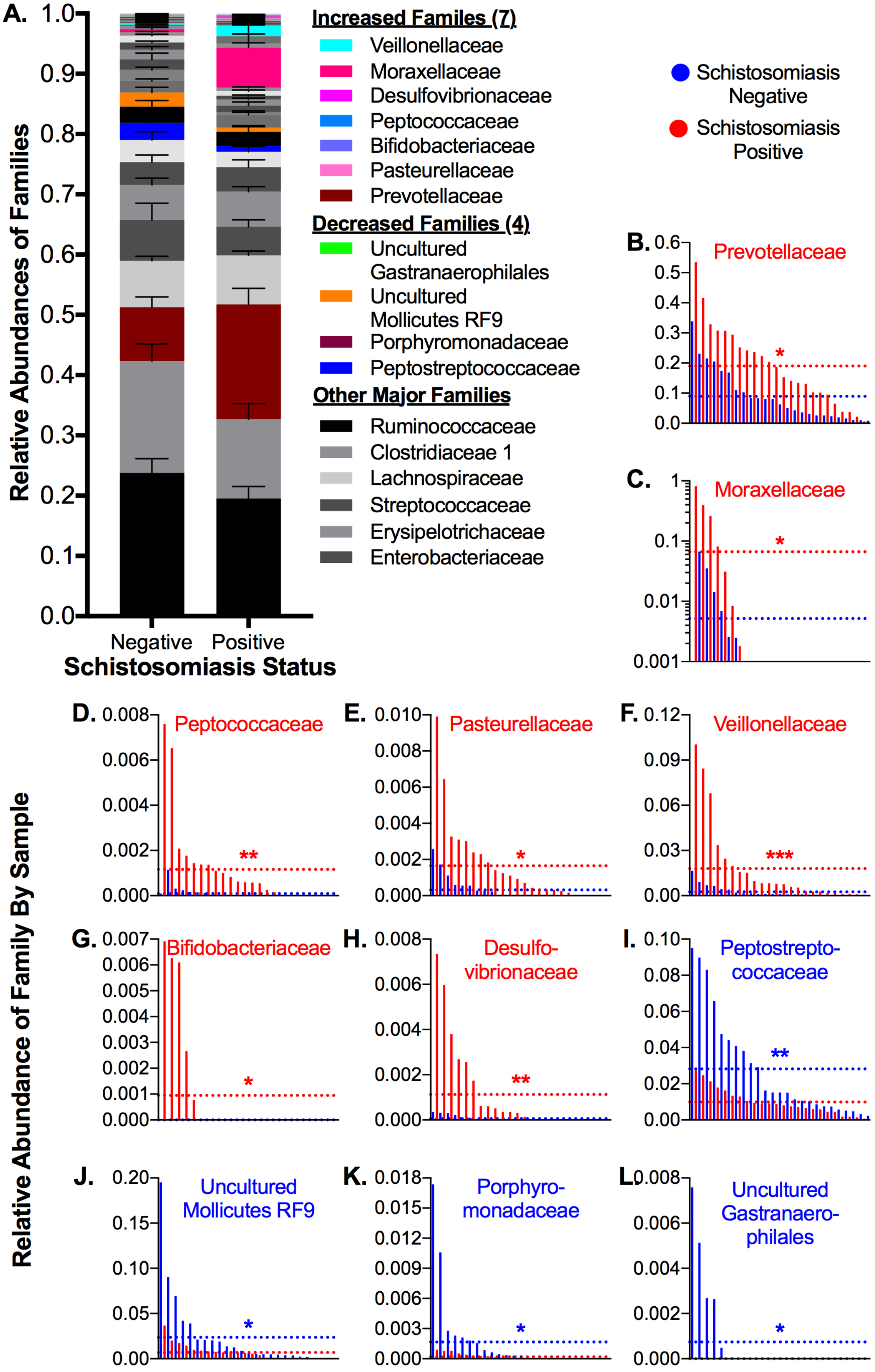

Figure S5: Fold Changes of Differentially-Abundant Lineages Between Schistosomiasis-positive and -negative Groups.
(A) *Desulfovibrio* lineage (B) *Acinetobacter* and *Haemophilus* lineage (C) *Megasphaera* and *Dialister* lineage (D) *Peptococcus* and Peptostreptococcaceae *incertae sedis* lineage. Statistics: Wald test of differential abundance through DESeq2 package in R, * $p<0.05$, ** $p<0.01$, *** $p<0.001$, **** $p<0.0001$, error bars indicate standard error of $\log_2$(Fold Change).

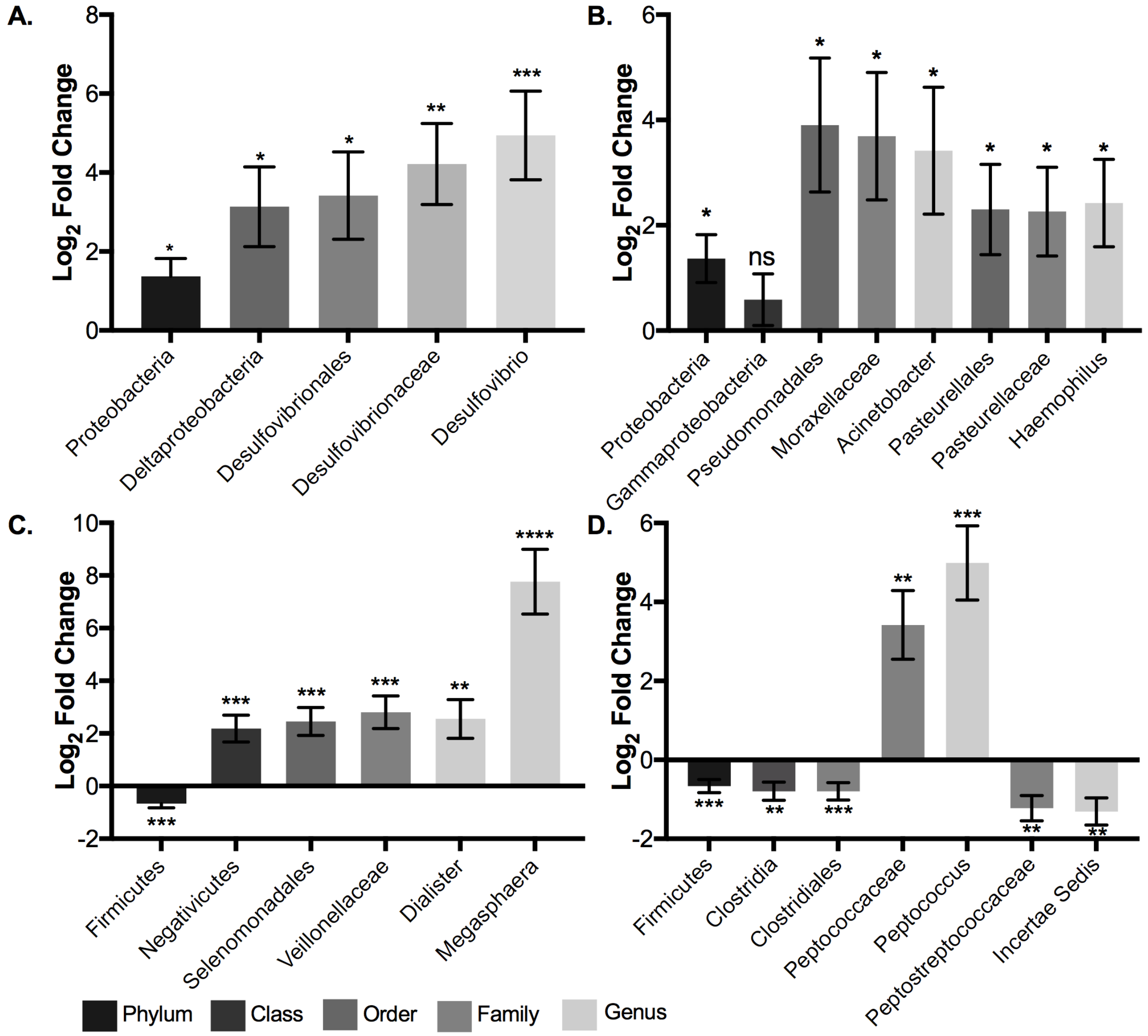

Figure S6: PCR to Detect Helminth DNA in Fecal Samples.
(A) PCR for *S. mansoni* and *S. haematobium*. (B) PCR for *Ascaris* spp. (C) PCR for *Ancyclostoma* spp. (D) PCR for *Necator americanus*. (E) PCR for *Trichuris trichiura*. Lane Labels: (1) Positive Control gDNA, (2) Pooled Schistosomiasis-Negative Fecal DNA, (3) Pooled Schistosomiasis-Positive Fecal DNA. All gels were photographed using the auto-exposure setting of the Gel Doc EZ-Imager (BioRad). All images are cropped to focus on the band(s) of interest, but full-length gels can be found in Figure S7.

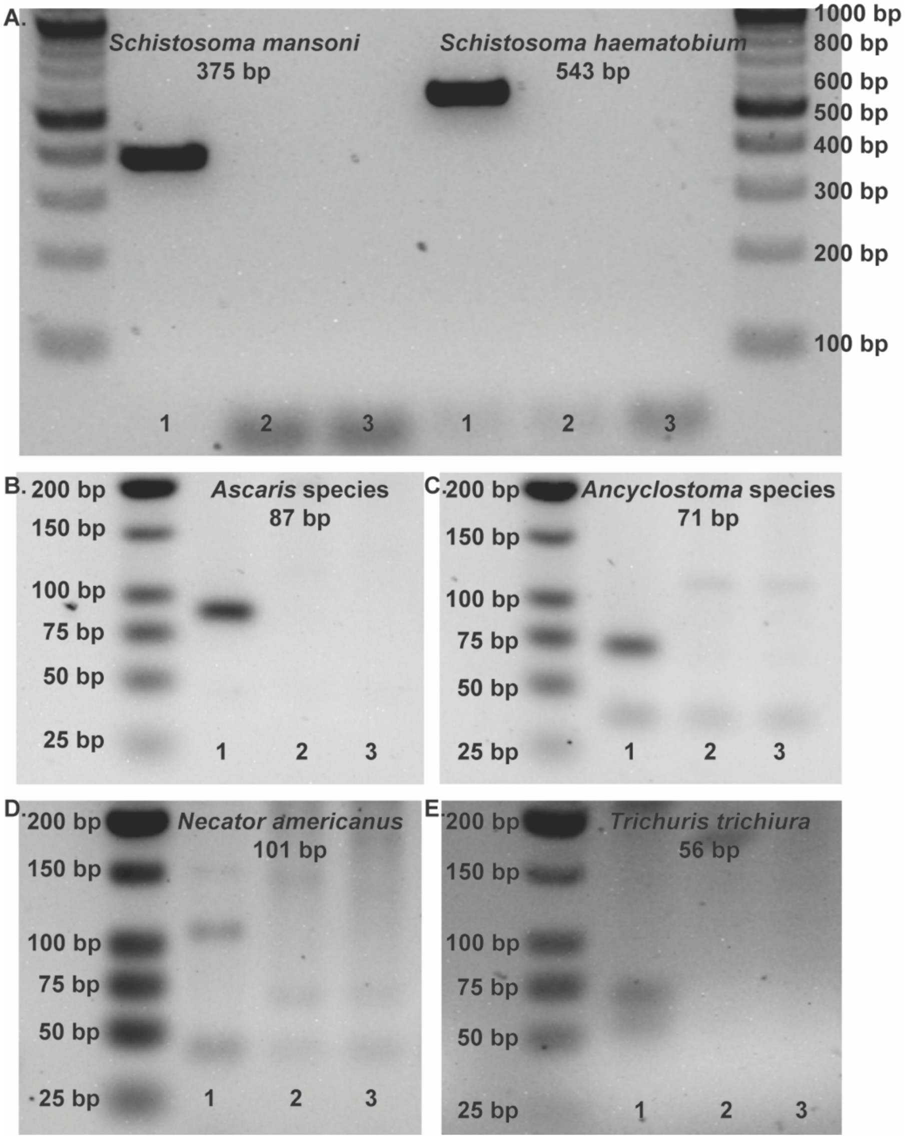

Figure S7: Full-length gels of helminth PCRs from Figure S6.
(A) Uncropped gel of PCR for *S. mansoni* and *S. haematobium*. (B) Uncropped gel of PCR for *Ascaris* spp. (C) Uncropped gel of PCR for *Ancyclostoma* spp. (D) Uncropped gel of PCR for *N. americanus*. (E) Uncropped gel of PCR for *T. trichiura*.

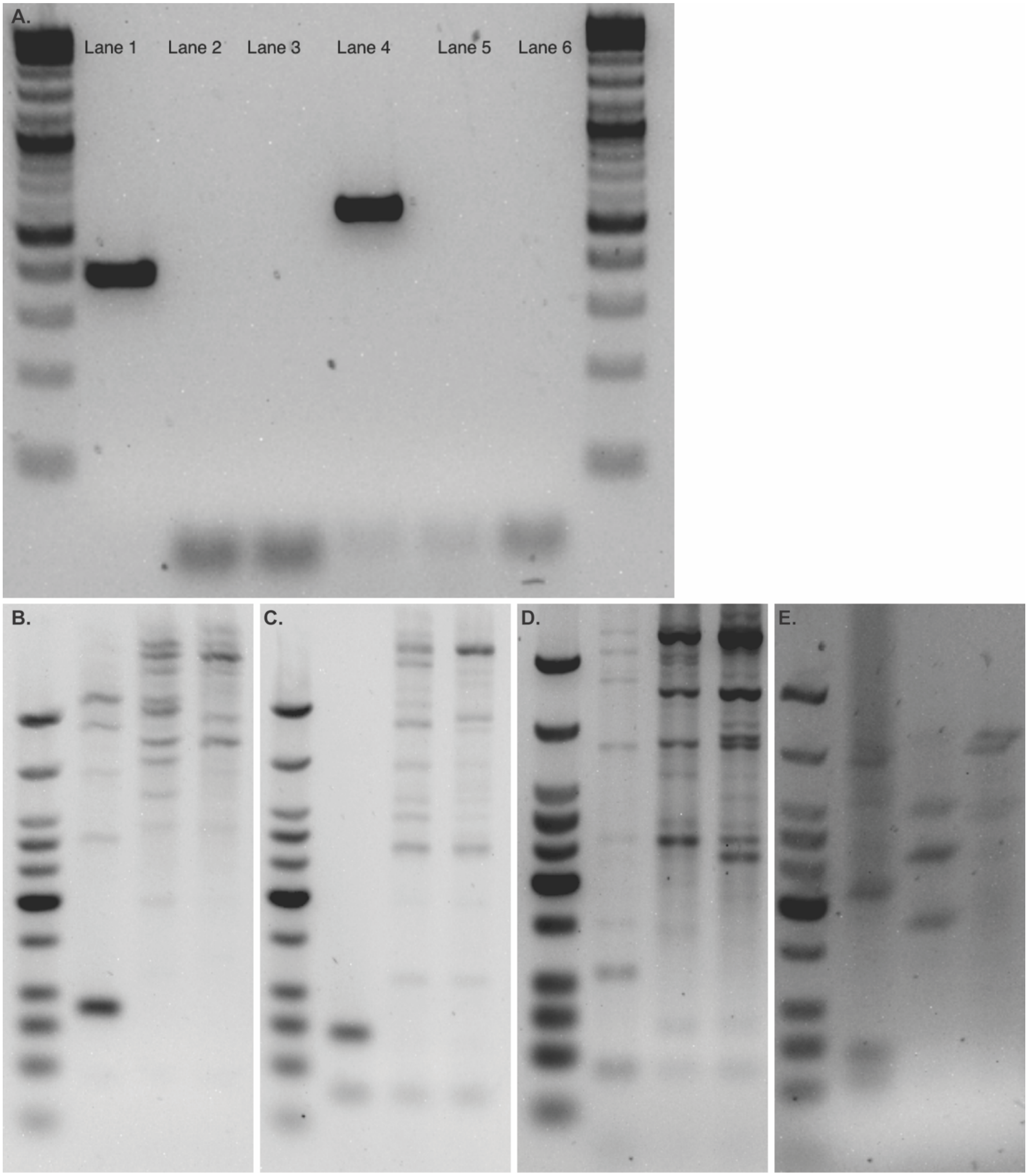

Table S1: Demographic Data on Study Participants.
This table includes subject number, biological sex, age, schistosomiasis infection status, and urine egg counts per 10 mL for infected participants. Subject 42 (indicated with *) was not included in analysis due to low-quality amplification of 16S rDNA.

Subject	Sex	Age	Schistosomiasis	Eggs	Subject	Sex	Age	Schistosomiasis	Eggs
1	Male	11	Negative	0	26	Male	12	Positive	20
2	Male	11	Negative	0	27	Male	11	Positive	250
3	Male	12	Negative	0	28	Male	11	Positive	20
4	Male	11	Negative	0	29	Male	13	Positive	10
5	Male	11	Negative	0	30	Male	13	Positive	40
6	Male	12	Negative	0	31	Male	12	Positive	380
7	Male	13	Negative	0	32	Male	11	Positive	100
8	Male	12	Negative	0	33	Male	11	Positive	10
9	Male	11	Negative	0	34	Male	15	Positive	20
10	Male	11	Negative	0	35	Male	11	Positive	80
11	Male	12	Negative	0	36	Male	11	Positive	110
12	Male	12	Negative	0	37	Male	12	Positive	20
13	Male	12	Negative	0	38	Male	13	Positive	90
14	Male	12	Negative	0	39	Male	15	Positive	30
15	Male	15	Negative	0	40	Male	11	Positive	10
16	Male	11	Negative	0	41	Male	15	Positive	40
17	Male	11	Negative	0	42*	Male	11	Positive	100
18	Male	15	Negative	0	43	Male	15	Positive	20
19	Male	15	Negative	0	44	Male	13	Positive	20
20	Male	13	Negative	0	45	Male	13	Positive	30
21	Female	12	Negative	0	46	Male	15	Positive	50
22	Female	12	Negative	0	47	Male	12	Positive	50
23	Female	11	Negative	0	48	Female	12	Positive	50
24	Female	11	Negative	0	49	Female	11	Positive	30
25	Female	11	Negative	0	50	Female	11	Positive	30

Table S2: Lifestyle Data on Study Participants.

This table includes the source of drinking water, weekly exposure to river water, and maternal occupation of all subjects. Subjects did not significantly differ on any metric by infection group, as analyzed by chi-square analysis (p = 0.732, p = 0.340, p = 0.958). To meet the requirements for chi-square analysis, the “tap” and “river” responses were combined and the “housewife” and “none” responses were combined in the contingency tables for “Drinking Water Source” and “Maternal Occupation”, respectively.

Schistosomiasis-Negative				Schistosomiasis-Positive			
Subject	Drinking Water Source	Weekly River Contact	Maternal Occupation	Subject	Drinking Water Source	Weekly River Contact	Maternal Occupation
1	Tap	Once	Farmer	26	Well	Once	None
2	Tap	Never	Farmer	27	Well	Once	Office worker
3	Well	Once	Farmer	28	Tap	Twice	None
4	Well	Once	Farmer	29	Well	Twice	Farmer
5	Well	Daily	Farmer	30	Well	Never	None
6	Well	Once	Housewife	31	Well	Once	Farmer
7	Well	Once	None	32	Tap	Twice	None
8	Well	Twice	None	33	Well	Twice	Farmer
9	Well	Daily	Office worker	34	Tap	Daily	None
10	Well	Daily	None	35	Well	Once	Farmer
11	Well	Once	Farmer	36	Well	Once	Farmer
12	Well	Once	Farmer	37	Well	Once	None
13	Well	Never	Office worker	38	Well	Never	None
14	Well	Daily	None	39	Well	Once	Farmer
15	Well	Twice	None	40	Tap	Never	Farmer
16	Tap	Daily	None	41	Well	Never	Office worker
17	Well	Once	Farmer	42	Well	Once	None
18	Well	Once	Farmer	43	River	Daily	Farmer
19	Tap	Once	None	44	Well	Twice	Farmer
20	Well	Twice	Farmer	45	Well	Twice	None
21	Well	Daily	Farmer	46	Well	Twice	None
22	Tap	Never	None	47	Well	Daily	None
23	Tap	Never	None	48	Well	Once	Farmer
24	Well	Never	None	49	Well	Once	None
25	Well	Daily	None	50	Well	Once	None
Summary (Negative)	19 Well<br>6 Tap<br>0 River	5 Never<br>10 Once<br>3 Twice<br>7 Daily	11 Farmer<br>1 Housewife<br>2 Office Worker<br>11 None	Summary (Positive)	20 Well<br>4 Tap<br>1 River	4 Never<br>11 Once<br>7 Twice<br>3 Daily	10 Farmer<br>0 Housewife<br>2 Office Worker<br>13 None

Table S3: Differentially-abundant KEGG Pathways.
This table shows the two KEGG pathways that were significantly enriched in schistosomiasis-positive predicted metagenomes, as well as significantly-enriched orthologs within those pathways.

KEGG Pathway	Included Orthologs	FDR
Atrazine degradation	ureC (urease subunit alpha)<br>ureB (urease subunit beta)<br>ureA (urease subunit gamma)	0.000337
Arginine and proline metabolism	prdB (D-proline reductase)<br>prdF (proline racemase)	0.0156

Table S4: Differentially-abundant KEGG Orthologs.
This table shows KEGG orthologs that were differentially abundant in the schistosomiasis-positive and -negative groups. Orthologs in bold are members of significantly enriched pathways. Fold changes reflect the abundance in schistosomiasis-positive relative to -negative subjects.

KEGG Ortholog	Name	Fold Change	FDR
K07006	uncharacterized protein	6.825808785	9.31E-05
K05346	deoR; deoxyribonucleoside regulator	6.93453911	9.31E-05
K03929	pnbA; para-nitrobenzyl esterase	5.402398268	0.0012918
K07454	putative restriction endonuclease	5.698570258	0.0021704
K01501	nitrilase	5.716769016	0.0021704
K00019	bdh; 3-hydroxybutyrate dehydrogenase	4.781953164	0.0023841
K08365	merR; MerR family transcriptional regulator, mercuric resistance operon regulatory protein	4.478141143	0.0051743
K07276	uncharacterized protein	5.018652309	0.0051743
**K10794**	**prdB; D-proline reductase (dithiol)**	**4.987787494**	**0.0051743**
K10811	thiamine pyridinylase	4.987787494	0.0051743
K20626	lcdA; lactoyl-CoA dehydratase subunit alpha	4.987787494	0.0051743
K20627	lcdB; lactoyl-CoA dehydratase subunit beta	4.987787494	0.0051743
**K01777**	**prdF; proline racemase**	**4.927650723**	**0.0059194**
K18923	stbD; antitoxin StbD	4.322601978	0.010191
K13928	mdcR; LysR family transcriptional regulator, malonate utilization transcriptional regulator	4.250698698	0.011485
K07267	oprB; porin	4.48124623	0.011485
K00529	hcaD; 3-phenylpropionate/trans-cinnamate dioxygenase ferredoxin reductase component	2.901904416	0.012016
K00480	salicylate hydroxylase	2.91925452	0.012272
K05819	mhpT; MFS transporter, AAHS family, 3-hydroxyphenylpropionic acid transporter	2.870893873	0.01367
**K01428**	**ureC; urease subunit alpha**	**3.343594748**	**0.01367**
K12542	lapC; membrane fusion protein, adhesin transport system	2.841987088	0.01367
K05710	hcaC; 3-phenylpropionate/trans-cinnamate dioxygenase ferredoxin component	2.841002301	0.01367
**K01430**	**ureA; urease subunit gamma**	**3.428315777**	**0.01367**
K02077	ABC.ZM.S; zinc/manganese transport system substrate-binding protein	2.928780351	0.013924
K03188	ureF; urease accessory protein	3.311993864	0.013924
K03189	ureG; urease accessory protein	3.311993864	0.013924
K03190	ureD, ureH; urease accessory protein	3.311075712	0.013924
K13818	mobAB; molybdopterin-guanine dinucleotide biosynthesis protein	4.304662112	0.013924
K03187	ureE; urease accessory protein	3.305113922	0.013924
**K01429**	**ureB; urease subunit beta**	**3.333181782**	**0.026514**
K01692	paaF, echA; enoyl-CoA hydratase	3.966317037	0.030301
K11103	dctA; aerobic C4-dicarboxylate transport protein	2.571303666	0.030301
K08728	nucleoside deoxyribosyltransferase	3.963019318	0.030301
K07783	uhpC; MFS transporter, OPA family, sugar phosphate sensor protein	0.413769727	0.030301
K00004	BDH, butB; (R,R)-butanediol dehydrogenase / meso-butanediol dehydrogenase / diacetyl reductase	3.043222884	0.04899

Table S5: Primers and Conditions Used for PCR Detection of Helminth DNA in Fecal Samples.

Organism/Gene	Primers	Cycle Conditions	Positive Control DNA
Schistosoma mansoni Cox1	FWD: TTTTTTGGTCATCCTGAGGTGTAT REV: TGCAGATAAAGCCACCCCTGTG	98C for 30 seconds 61C for 45 seconds 72C for 1 minute	*Schistosoma mansoni*, adult worm, male and female genomic DNA (mixed) (BEI)
Schistosoma haematobium Cox1	FWD: TTTTTTGGTCATCCTGAGGTGTAT REV: TGATAATCAATGACCCTGCAATAA	98C for 30 seconds 64C for 45 seconds 72C for 1 minute	*Schistosoma haematobium*, adult worm, male and female genomic DNA (mixed) (BEI)
Ascaris spp. ITS1	FWD: GTAATAGCAGTCGGCGGTTTCTT REV: GCCCAACATGCCACCTATTC	98C for 10 seconds 60C for 10 seconds 72C for 15 seconds	*Ascaris lumbricoides* genomic DNA (Williams lab)
Ancyclostoma spp. ITS1	FWD: GAATGACAGCAAACTCGTTGTTG REV: ATACTAGCCACTGCCGAAACGT	98C for 10 seconds 60C for 10 seconds 72C for 15 seconds	*Ancyclostoma duodenale* genomic DNA (Williams lab)
Necator americanus ITS2	FWD: CTGTTTGTCGAACGGTACTTGC REV: ATAACAGCGTGCACATGTTGC	98C for 10 seconds 57C for 10 seconds 72C for 15 seconds	*Necator americanus* genomic DNA (Williams lab)
Trichuris trichiura ITS1	FWD: TCCGAACGGCGGATCA REV: CTCGAGTGTCACGTCGTCCTT	98C for 10 seconds 57C for 10 seconds 72C for 15 seconds	*Trichuris trichuria* genomic DNA (Williams lab)

Table S6: Primers Used for Amplicon Generation.

This table includes the standard 5' Illumina Adapter, Pad, Linker, and 515F primer segments as well as the variable barcodes used for sample identification and demultiplexing. It also includes the 806R primer, with the associated Pad, Linker, and Adapter segments. Primer design was obtained from the Earth Microbiome Project protocols.

Primer Structure	
Forward Primer	5' Illumina Adapter – Barcode – Pad – Linker – 515FB Primer<br>AATGATACGGCGACCACCGAGATCTACACGCT-BARCODE-TATGGTAATT-GT-GTGYCAGCMGCCGCGGTAA
Reverse Primer	CAAGCAGAAGACGGCATACGAGAT-AGTCAGCCAG-CC-GGACTACNVGGGTWTCTAAT

Subject	Barcode Sequence	Subject	Barcode Sequence
AR001	AGCCTTCGTCGC	AR026	CGGGACACCCGA
AR002	TCCATACCGGAA	AR027	CTGTCTATACTA
AR003	AGCCCTGCTACA	AR028	TATGCCAGAGAT
AR004	CCTAACGGTCCA	AR029	CGTTTGGAATGA
AR005	CGCGCCTTAAAC	AR030	AAGAACTCATGA
AR006	TATGGTACCCAG	AR031	TGATATCGTCTT
AR007	TACAATATCTGT	AR032	CGGTGACCTACT
AR008	AATTTAGGTAGG	AR033	AATGCGCGTATA
AR009	GACTCAACCAGT	AR034	CTTGATTCTTGA
AR010	GCCTCTACGTCG	AR035	GAAATCTTGAAG
AR011	ACTACTGAGGAT	AR036	GAGATACAGTTC
AR012	AATTCACCTCCT	AR037	GTGGAGTCTCAT
AR013	CGTATAAATGCG	AR038	ACCTTACACCTT
AR014	ATGCTGCAACAC	AR039	TAATCTCGCCGG
AR015	ACTCGCTCGCTG	AR040	ATCTAGTGGCAA
AR016	TTCCTTAGTAGT	AR041	ACGCTTAACGAC
AR017	CGTCCGTATGAA	AR042	TACGGATTATGG
AR018	ACGTGAGGAACG	AR043	ATACATGCAAGA
AR019	GGTTGCCCTGTA	AR044	CTTAGTGCAGAA
AR020	CATATAGCCCGA	AR045	AATCTTGCGCCG
AR021	GCCTATGAGATC	AR046	AGGATCAGGGAA
AR022	CAAGTGAAGGGA	AR047	AATAACTAGGGT
AR023	CACGTTTATTCC	AR048	TATTGCAGCAGC
AR024	TAATCGGTGCCA	AR049	TGATGTGCTAAG
AR025	TGACTAATGGCC	AR050	GTAGTAGACCAT

Table S7: Read Depths by Sample.

Read depths did not differ significantly by sample.

Sample (Negative)	Reads	Sample (Positive)	Reads
AR001	177382	AR026	25868
AR002	34580	AR027	29106
AR003	109962	AR028	43414
AR004	46723	AR029	36513
AR005	29127	AR030	45852
AR006	36463	AR031	42730
AR007	43240	AR032	33965
AR008	39282	AR033	13797
AR009	50435	AR034	29927
AR010	35716	AR035	43143
AR011	29119	AR036	40509
AR012	35924	AR037	27851
AR013	39648	AR038	31072
AR014	40182	AR039	25288
AR015	22118	AR040	31453
AR016	41157	AR041	27389
AR017	27675	AR042	14 (not analyzed)
AR018	38916	AR043	27188
AR019	25422	AR044	41881
AR020	48118	AR045	31704
AR021	27700	AR046	57774
AR022	36180	AR047	31301
AR023	39116	AR048	32661
AR024	33414	AR049	39150
AR025	35564	AR050	26365

Table S8: Genus-specific Primers and Cycle Conditions Used for qPCR Confirmation of Genus Changes.

Genus	PCR Primers	Cycle Conditions
Megasphaera	Forward: AGAGACTGCCGCAGACAATGCGGAGG<br>Reverse: TTTGGGGTTTGCTCCGGATCGCTCCTT	98C for 10 seconds<br>74C for 30 seconds<br>(2-step)
Dialister	Forward: GGAAACTGGGAAGCTGGAGTATC<br>Reverse: TTAATCTTGCGATCGTACTTCCCAGG	98C for 10 seconds<br>66C for 10 seconds<br>72C for 10 seconds
Peptococcus	Forward: AGTGGGGAATAACAGTGAGAAATCA<br>Reverse: TCTCTTGGATGAGGACAGAGTTTT	98C for 10 seconds<br>65C for 10 seconds<br>72C for 10 seconds
Prevotella	Forward: CTATGGGTTGTAAACTGCT<br>Reverse: ACATTTCACAACACGCTTA	98C for 10 seconds<br>56C for 10 seconds<br>72C for 10 seconds
Olsenella	Forward: GGTGAAGCGGCGGAGACGCCGTGGCCG<br>Reverse: GGTCTCGCATGGGTGCCCGGCCGAA	98C for 10 seconds<br>74C for 30 seconds<br>(2-step)
Alloprevotella	Forward: AGAAAAAGGACCGGCTAATT<br>Reverse: AGTTTCAACTGCA	98C for 10 seconds<br>59C for 10 seconds<br>72C for 10 seconds
Haemophilus	Forward: ATAACTACGGGAAACTGTAGCTAAT<br>Reverse: ACACCTCACTTAAGTCACCG	98C for 10 seconds<br>58C for 10 seconds<br>72C for 10 seconds
Parabacteroides	Forward: ACCCGGGTTTGAACG<br>Reverse: CAGCTTACGCTGGCAGTC	98C for 10 seconds<br>60C for 10 seconds<br>72C for 10 seconds
Subdoligranulum	Forward: GGCATCGGATTGAGGGAAA<br>Reverse: TGTCTCAGTCCCAATGTGGC	98C for 10 seconds<br>61C for 10 seconds<br>72C for 10 seconds
Total 16S	Forward: CCAGCAGCYGCGGTAAN<br>Reverse: GGACTACHVGGGTWTCTAATCC	98C for 10 seconds<br>55C for 10 seconds<br>72C for 10 seconds

S1 Data: Per-sample Relative Abundance Data.
Tab 1 includes phylum-level data, Tab 2 includes class-level data, Tab 3 includes order-level data, Tab 4 includes family-level data, and Tab 5 includes genus-level data.
